# Naming fMRI predicts the effect of temporal lobe resection on language decline

**DOI:** 10.1002/acn3.50911

**Published:** 2019-10-02

**Authors:** Karin Trimmel, Louis A. van Graan, Gloria G. Gonzálvez, Anja Haag, Lorenzo Caciagli, Sjoerd B. Vos, Silvia Bonelli, Meneka Sidhu, Pamela J. Thompson, Matthias J. Koepp, John S. Duncan

**Affiliations:** ^1^ Epilepsy Society MRI Unit Chalfont Centre for Epilepsy Chalfont St Peter SL9 0LR United Kingdom; ^2^ Department of Clinical and Experimental Epilepsy UCL Queen Square Institute of Neurology Queen Square London WC1N 3BG United Kingdom; ^3^ Department of Neurology Medical University of Vienna A‐1090 Vienna Austria; ^4^ Centre for Medical Image Computing University College London London United Kingdom

## Abstract

**Objective:**

To develop language functional MRI (fMRI) methods that accurately predict postsurgical naming decline in temporal lobe epilepsy (TLE).

**Methods:**

Forty‐six patients with TLE (25 left) and 19 controls underwent two overt fMRI paradigms (auditory naming and picture naming, both with active baseline conditions) and one covert task (verbal fluency). Clinical naming performance was assessed preoperatively and 4 months following anterior temporal lobe resection. Preoperative fMRI activations were correlated with postoperative naming decline. Individual laterality indices (LI) were calculated for temporal (auditory and picture naming) and frontal regions (verbal fluency) and were considered as predictors of naming decline in multiple regression models, along with other clinical variables (age at onset of seizures, preoperative naming scores, hippocampal volume, age).

**Results:**

In left TLE patients, activation of the left posterior inferior temporal gyrus during auditory naming and activation of left fusiform gyrus during picture naming were related to greater postoperative naming decline. Activation LI were the best individual predictors of naming decline in a multivariate regression model. For picture naming, an LI of higher than 0.34 gave 100% sensitivity and 92% specificity (positive predictive value (PPV) 91.6%). For auditory naming, a temporal lobe LI higher than 0.18 identified all patients with a clinically significant naming decline with 100% sensitivity and 58% specificity (PPV: 58.3%). No effect was seen for verbal fluency.

**Interpretation:**

Auditory and picture naming fMRI are clinically applicable to predict postoperative naming decline after left temporal lobe resection in individual patients, with picture naming being more specific.

## Introduction

Anterior temporal lobe resection (ATLR) is an effective treatment option for medically refractory temporal lobe epilepsy (TLE), leading to postoperative seizure freedom in up to 80% of patients. Naming decline following language‐dominant ATLR is a relevant concern, affecting 30–50% of patients following language‐dominant ATLR. Language fMRI is used during presurgical assessment as a predictor of a reduction in naming capacity^1^, ^2^ and to date, has had high sensitivity, but relatively low specificity.^1^

Most clinically used language fMRI paradigms, for example, verbal fluency or verb generation tasks, primarily activate frontal lobe language regions,^1^, ^3^, ^4^ and not the temporal lobe, that is most affected by reorganization in TLE, and resected in ATLR.^5^ A recent practice guideline summary recommended that language fMRI is considered to predict postsurgical language outcome after ATLR (Level C), but emphasized the need for future research in the field.^6^

We recently showed that auditory and picture naming fMRI tasks, when used with active baseline conditions (reversed speech and scrambled pictures, respectively), reliably activate posterior and basal temporal lobe regions, and are strongly related to clinical naming performance.^7^ To investigate the possibility to predict postoperative naming decline in TLE, we compared auditory and picture naming to standard verbal fluency fMRI tasks in a cohort of patients with left TLE (LTLE) and right TLE (RTLE) undergoing temporal lobe surgery.

We hypothesized that:

1. Auditory and picture naming fMRI activations in the posterior basal temporal lobe are related to greater naming decline after temporal lobe surgery.
2. Stronger lateralization of naming fMRI activations to the to‐be‐resected temporal lobe are related to greater naming decline.
3. Individual laterality indices (LI) of naming fMRI task activations will be predictive of a clinically relevant naming decline in the individual patient, particularly after left temporal lobe resection.

## Methods

### Subjects

Forty‐six medically refractory TLE patients (20 females; age range 19–58 years) consecutively undergoing epilepsy surgery at the National Hospital for Neurology and Neurosurgery (NHNN) in London, UK, between 2013 and 2017 were enrolled in the study, 25 with LTLE and 21 with RTLE. Nineteen healthy participants (10 females, age range 23–63 years) formed the control group. Exclusion criteria for all participants were nonfluency in written and spoken English, pregnancy, any contraindication to MRI, and inability to give informed consent. An additional exclusion criterion for TLE patients was history of a secondarily generalized tonic‐clonic seizure within 24h prior to the study. Demographic and clinical data are summarized in Table 1.

**Table 1:** Demographic and clinical data for LTLE patients, RTLE patients, and control subjects.

	Gender female/male	Handedness right/left	Age (years)	Age onset (years)	Duration(years)	CPS monthly	SGS monthly	Number AED	McKenna Naming score	Gender female/male
LTLE (*n* = 25)	10/15	21/4	37.5 ± 11.2	17.8 ± 10.7	13 (29)	4 (8.3)	0 (0)	3 (1.5)	14.9 ± 5.8*	96.9 ± 11.3‡
RTLE (*n* = 21)	10/11	19/2	38.0 ± 11.1	20.2 ± 14.7	16 (13.5)	5 (7.0)	0 (0.4)	2 (1.0)	17.0 ± 5.7	98.5 ± 14.7†
CTR (*n* = 19)	10/9	17/2	41.7 ± 11.5	n.a.	n.a.	n.a.	n.a.	n.a.	19.7 ± 4.9	110.0 ± 10.1

Age, age at onset of epilepsy, clinical naming scores, and estimated intellectual level (IQ) are shown as mean ± SD. Disease duration, seizure frequency (CPS, SGS), and number of AED are shown as median and IQR. AED, number of different antiepileptic drugs taken per day at time of scan; CPS, complex partial seizures; CTR, control subjects; IQ, estimated intellectual level; IQR, interquartile range; LTLE, left temporal lobe epilepsy; RTLE, right temporal lobe epilepsy; SD, standard deviation; SGS, secondarily generalized seizures.

* Naming score LTLE < CTR *P* = 0.01.

^†^ IQ RTLE < CTR *P* = 0.02.

^‡^ IQ LTLE < CTR *P* = 0.002.

All patients had preoperative prolonged interictal and ictal EEG video telemetry that confirmed and lateralized temporal seizure onset zones (ipsilateral in patients with structural brain lesions). All patients underwent structural MRI at 3T, identifying hippocampal sclerosis (HS) in 24 patients (14 left/10 right), dysembryoplastic neuroepithelial tumor (DNET) in 11 (7 left/4 right), cavernoma in five (3 left/ 2 right), focal cortical dysplasia in two (both right), low grade glioma in two (1 left/1 right), dual pathology (FCD and HS) in one (right), and encephalocele in one (right). Seventy‐three percent of patients underwent standard en‐bloc ATLR, including resection of the hippocampus. Twenty‐seven percent of patients underwent a temporal lobe lesionectomy. All participants were fluent in written and spoken English. Handedness was determined using the Edinburgh Hand Preference Inventory.^8^ The distribution of age was comparable among the three groups (one‐way ANOVA, *P* > 0.05; Table 1). The two patient groups did not differ for age of onset of epilepsy, disease duration, seizure frequency, or number of AEDs (independent samples two‐tailed *T*‐test, respectively Kruskal–Wallis test; Table 1).

### Standard protocol approvals, registrations, and patient consents

The study was approved by the NHNN and UCL Institute of Neurology Joint Research Ethics Committee. Written informed consent was obtained from all participants.

### Neuropsychological tests

All subjects underwent neuropsychological testing prior to scanning to provide a measure of their linguistic proficiency. The measures employed were standardized clinical tests that form part of the pre‐ and postsurgical neuropsychological evaluations of TLE patients. Naming was assessed using the McKenna Graded Naming Test (GNT),^9^ which consists of 30 line drawings of objects and animals, placed in order of difficulty. The total number of correctly named items is the performance indicator.^9^ The GNT is a long‐standing, widely used test with excellent intertrial reliability (0.96).^10^ A reliable change index (RCI) of 3.7 points has been suggested for performance gains, and an RCI of −1.5 points indicates decline.^10^ In the present study, the GNT was performed preoperatively and 4 months postoperatively, and a decline of ≥4 items was considered clinically significant.^1^, ^5^

Controls were also retested approximately 8 months after the initial investigation, which matched to the interval between investigations in patients due to a median time lag of 4 months between the fMRI investigation and epilepsy surgery in patients. Intellectual level was derived from performance on the National Adult Reading Test (NART).^11^

### Magnetic resonance data acquisition

Please refer to the Appendix for details on MRI data acquisition.

### Language paradigms

We employed two overt language tasks, auditory naming (AN), picture naming (PN), and a covert verbal fluency (VF) paradigm as described previously.^7^, ^12^, ^13^, ^14^ Subjects responded to visual and auditory stimuli presented via a magnetic resonance‐compatible screen viewed through a mirror^1^, ^7^, ^12^ and a compatible audio system (headphone and microphone devices).

AN sessions consisted of five cycles of alternating 30‐s activation blocks and two control blocks of 15‐sec each, comprising reversed speech (AR) and crosshair fixation. During the activation phase, subjects were asked to name aloud objects and animals from their auditory description. Participants were instructed to count aloud “one, two” during AR and to rest with eyes open during crosshair fixation.

PN sessions involved five cycles of visually presented stimuli, each consisting of alternating 30‐sec activation blocks and three control blocks of 15‐sec each, comprising scrambled pictures (SPc), blurred cartoon faces (F), and crosshair fixation. During the activation phase, participants were instructed to name aloud black and white line drawings of everyday objects and animals. Subjects were instructed to count aloud “one, two” in response to SPc and F, and to rest with eyes open during crosshair fixation.

VF comprised a blocked experimental design with alternating 30‐s activation blocks and 30‐s of crosshair fixation over 5 min.^7^, ^12^ During the activation phase, subjects were asked to covertly generate different words beginning with a visually presented letter (A, S, W, D, and E) and to rest with eyes open during crosshair fixation.

Prior to scanning, each subject was given detailed explanations with examples to ensure test instructions were fully understood. We recorded all tasks with an external microphone inside the scanner. All study participants successfully performed >80% on the overt functional MRI tasks (AN and PN). Due to technical problems with the audio and visual presentation systems, AN could not be acquired in three LTLE patients. Because of poor fMRI data quality, AN data of two patients as well as VF data of one patient had to be excluded from the analyses.

### fMRI data analysis

Imaging data were analyzed using Statistical Parametric Mapping 8 (http://www.fil.ion.ucl.ac.uk/spm/). The imaging time series of each subject was realigned, normalized into standard anatomical space using a scanner specific template (created from high‐resolution whole brain echo planar images of 30 healthy controls, 15 patients with left hippocampal sclerosis, and 15 patients with right hippocampal sclerosis) and smoothed with a Gaussian kernel of 8 mm full‐width at half‐maximum.

A two‐level random effects analysis was employed. In the first level, condition‐specific effects were estimated according to the general linear model^15^ for each subject. Regressors of interest were formed by convolving blocks of stimuli with the canonical hemodynamic response function for each of the conditions of interest, including motion parameters as confounds. Parameter estimates for regressors were calculated for each voxel. Three contrast images were generated for each subject within the three groups (LTLE, RTLE, CTR), comprising (1) auditory naming minus reversed speech (AN–AR), (2) picture naming minus scrambled pictures and faces (PN–(SPc + F)), and (3) VF. For the convenience of the reader, we refer to the contrast AN–AR as “auditory naming,” and the contrast PN–(SPc + F) as “picture naming,” and the contrast VF as “verbal fluency.”

These contrast images were used for the second‐level analysis. A one‐sample *t*‐test was used to examine group effects for task‐relevant activations and deactivations across the three groups. One‐way ANOVA was used to quantitatively assess statistical differences in activations and deactivations among groups (LTLE, RTLE, controls). Unless otherwise stated, we report peak‐level activations at a threshold of *P* < 0.05, corrected for multiple comparisons (family‐wise error rate [FWE]) across the whole brain. Estimated verbal IQ derived from performance on the NART^11^ was used as a covariate of no interest for all analyses. Due to a scanner upgrade in 2014, type of scanner was used as an additional covariate of no interest for all analyses.

#### Language dominance

Lateralization indices (LIs) were calculated to quantitatively assess hemispheric dominance for language,^16^ using the bootstrap method of the lateralization index toolbox implemented in SPM8^17^ on three spmT maps (corresponding to auditory naming, picture naming, and verbal fluency), based on anatomical masks comprising the bilateral anterior and posterior temporal lobe (superior, middle, inferior temporal gyrus and fusiform gyrus) and mesial temporal lobe structures (hippocampus, parahippocampal gyrus) for auditory naming and picture naming, and the inferior and middle frontal gyri for verbal fluency. The masks were created from the WFU PickAtlas in SPM8,^18^ in accordance with previous investigations.^7^, ^12^ According to the formula [LI = (L − R)/(L + R)], a positive LI indicates left hemispheric dominance and a negative index indicates right hemispheric lateralization. In line with standard practice in language fMRI research, we defined LI > +0.2 as left hemisphere dominant, bilateral as (−0.2 ≤ LI ≤ +0.2) and right hemisphere dominant (LI < −0.2).^12^, ^19^, ^20^

#### Relation of fMRI activation to naming decline

The relation of fMRI activation and the extent of naming decline was explored using naming decline as a regressor within multiple regression analysis models over the whole brain for each language task, masked with binarized group activation maps.^21^ All multiple regression activations are shown at an exploratory threshold of *P* < 0.001 uncorrected, in accordance with previous investigations.^7^, ^22^

### Statistical analysis

Statistical analyses were performed using SPSS 25.0 (Armonk, NY, USA). Between group comparisons were performed with one‐way ANOVA, independent samples two‐tailed *T*‐tests, and Kruskal–Wallis tests according to distribution of data. Correlations between LIs and naming decline scores in LTLE and RTLE patients were performed using Spearman correlation coefficients.

For each fMRI task, linear regression was applied to investigate the utility of language LIs and clinical variables (age, gender, age of onset of seizures, preoperative hippocampal volume, preoperative naming scores, surgery type) in predicting naming decline in LTLE and RTLE.

Receiver operating characteristic (ROC) curves were obtained and the area under the curve was calculated to identify the optimal LI cutoff value to identify patients with a significant naming decline (i.e., ≥4 points on the McKenna Graded naming Test^9^).

## Results

### Neuropsychological language performance

Groups differed significantly with respect to estimated intellectual level (F(2,62) = 6.99; *P* = 0.002) and naming scores (F(2,62) = 4.26; *P* = 0.02). Post hoc pairwise comparisons (Tukey HSD) indicated that mean estimated IQ was higher in controls than LTLE patients (*P* = 0.002) and RTLE patients (*P* = 0.01), while there was no significant difference between LTLE and RTLE patients (*P* = 0.90; Table 1). LTLE patients performed significantly less well on the out of scanner naming task than controls (*P* = 0.01), while there was no difference in naming scores between LTLE and RTLE patients (*P* = 0.40) or between RTLE patients and controls (*P* = 0.26, Table 1).

One LTLE patient performed in the impaired range (i.e. <1st centile) on the McKenna Graded Naming Test preoperatively and was therefore excluded from the prediction of naming decline analyses, as floor effects prevented identification of a postoperative decline.^1^ Data of one RTLE patient were excluded from the naming decline prediction analyses due to incomplete follow‐up.

Median naming decline 4 months postoperatively was 2 in LTLE (range +7 to −20 points) and 0 in RTLE (range +5 to −5 points). Clinically significant naming decline (≥4 points) was observed in 11 of 24 LTLE patients and 3 of 20 RTLE patients. Naming decline did not significantly differ between patients who were free of seizures postoperatively (75%) and those who continued to have seizures 4 months after surgery (25%; F(1,42) = 2.69, *P* = 0.77). None of the control subjects showed significant naming changes in the repeat investigation.

### Laterality indices

The distribution of lateralization of fMRI activations is displayed in Table 2. Kruskal–Wallis test for independent samples did not indicate a significant difference in the distribution of LIs across groups for auditory naming (H = 1.24; *P* = 0.54), picture naming (H = 0.10; *P* = 0.95), or verbal fluency (H = 1.20; *P* = 0.55).

**Table 2:** Distribution of lateralization of auditory naming, picture naming, and verbal fluency activations across the three groups (left TLE, right TLE, controls). Presented values indicate percentage of subjects showing left hemisphere dominance, bilateral distribution, or right hemisphere dominance. Left hemisphere dominance was defined as an LI of >+0.2, bilateral distribution as −0.2 ≤ LI ≤ +0.2), and right hemisphere dominance was defined as an LI < −0.2.

	Auditory naming			Picture naming			Verbal fluency		
	L	B	R	L	B	R	L	B	R
LTLE	65%	5%	30%	54%	17%	29%	75%	4%	21%
RTLE	67%	14%	19%	52%	19%	29%	91%	0%	9%
CTR	63%	5%	32%	58%	21%	21%	95%	0%	5%

L, left hemisphere dominance; B, bilateral representation; R, right hemisphere dominance; LTLE, left temporal lobe epilepsy; RTLE, right temporal lobe epilepsy; CTR, controls.

### fMRI results – main effects

**Figure 1:**
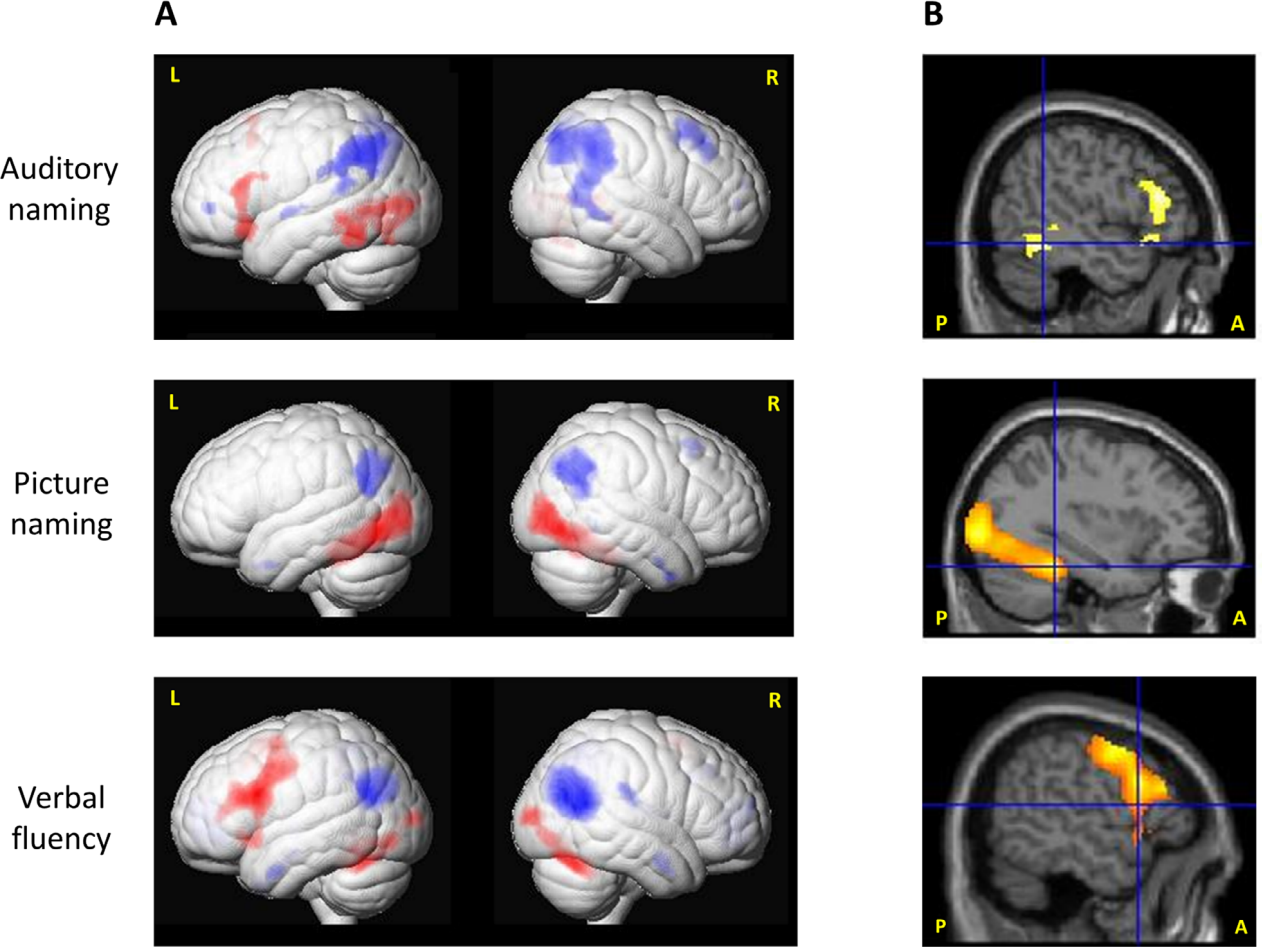
Main fMRI activation and deactivation. (A) Main activations (red) and deactivations (blue) across all three groups (LTLE, RTLE, controls) for auditory naming (upper row), picture naming (middle row), and verbal fluency (lower row) tasks shown rendered at *P* < 0.05, corrected for multiple comparisons (FWE). (B) Main fMRI activations across all three groups superimposed on sagittal slices. Crosshairs show left posterior inferior temporal gyrus fMRI activations for auditory naming, left fusiform gyrus activations for picture naming and left inferior frontal gyrus activations for verbal fluency. Auditory naming: Sagittal slices also show left inferior frontal activations. Visual naming: Sagittal slices also show left occipital activations. All activations are shown at a threshold of *P* < 0.05, voxel‐wise corrected for multiple comparisons (FWE) across the whole brain. Note: A = anterior; FWE = family‐wise error; L = left; LTLE = left temporal lobe epilepsy; P = posterior; R = right; RTLE = right temporal lobe epilepsy.

During auditory naming, main activations across the three groups were observed in the left posterior inferior temporal gyrus and posterior middle temporal gyrus, left fusiform gyrus, left inferior and superior frontal gyrus and supplementary motor region, left lingual gyrus, and left superior occipital gyrus (Fig. 1, Table S1). During picture naming, activations were seen in the left fusiform gyrus, left supplementary motor region, left middle occipital gyrus, bilateral cuneus, right inferior occipital gyrus, and right cerebellum (Fig. 1, Table S1). During verbal fluency, activations were seen in the left inferior frontal gyrus, left precentral gyrus and left supplementary motor area, right lingual gyrus, left inferior parietal lobule, left inferior occipital gyrus as well as bilateral middle occipital gyrus and cerebellum (Fig. 1, Table S1).

Task‐related deactivations across groups can be seen in Figure 1 and Table S1. Intergroup comparisons indicated no significant difference in activation or deactivation patterns among groups in all tasks.

### Relation of fMRI activations to naming decline

**Figure 2:**
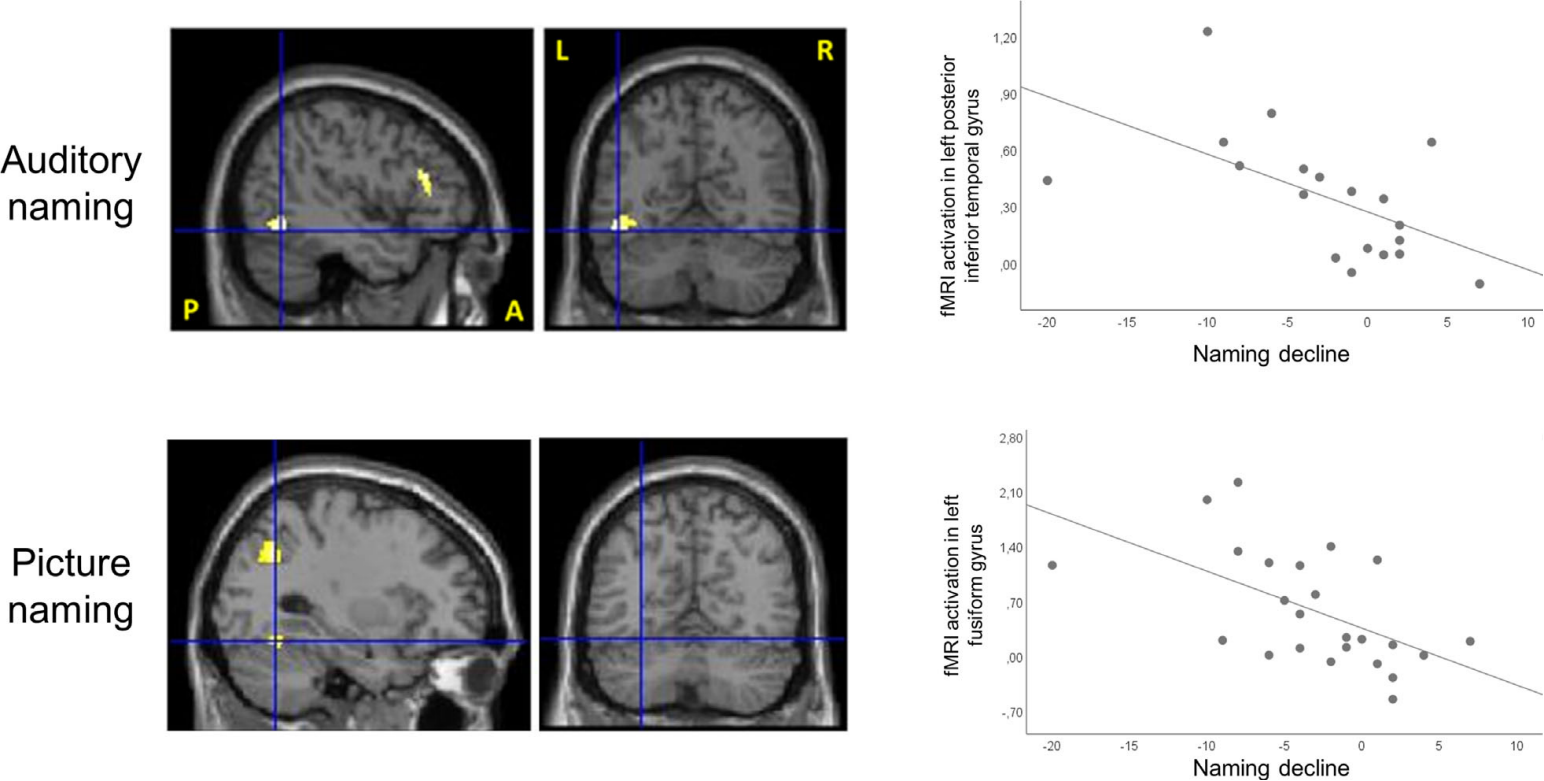
Correlation of fMRI activation (parameter estimates; *y* axis) with naming decline (McKenna Graded Naming Test change score; *x* axis) in left TLE. Activations are shown superimposed on coronal and sagittal images at *P* < 0.001, uncorrected, and the crosshair indicates the orthogonal slices. Stronger activations in the left inferior temporal gyrus during auditory naming (crosshair, upper row), and in the left fusiform gyrus during picture naming (crosshair, lower row), were associated with greater naming decline. Note: A = anterior; L = left; LTLE = left temporal lobe epilepsy; P = posterior; R = right.

In LTLE patients, stronger auditory naming activations in the left posterior inferior temporal gyrus and left inferior frontal gyrus as well as picture naming activations in the left fusiform gyrus, left middle occipital gyrus and right cerebellum were related to greater decline of naming scores (Fig. 2, Table S2). Verbal fluency activations were not related to naming decline in LTLE. In RTLE patients, activation during none of the tasks showed a relation to naming decline.

### Individual fMRI lateralization indices as predictors of naming decline

**Figure 3:**
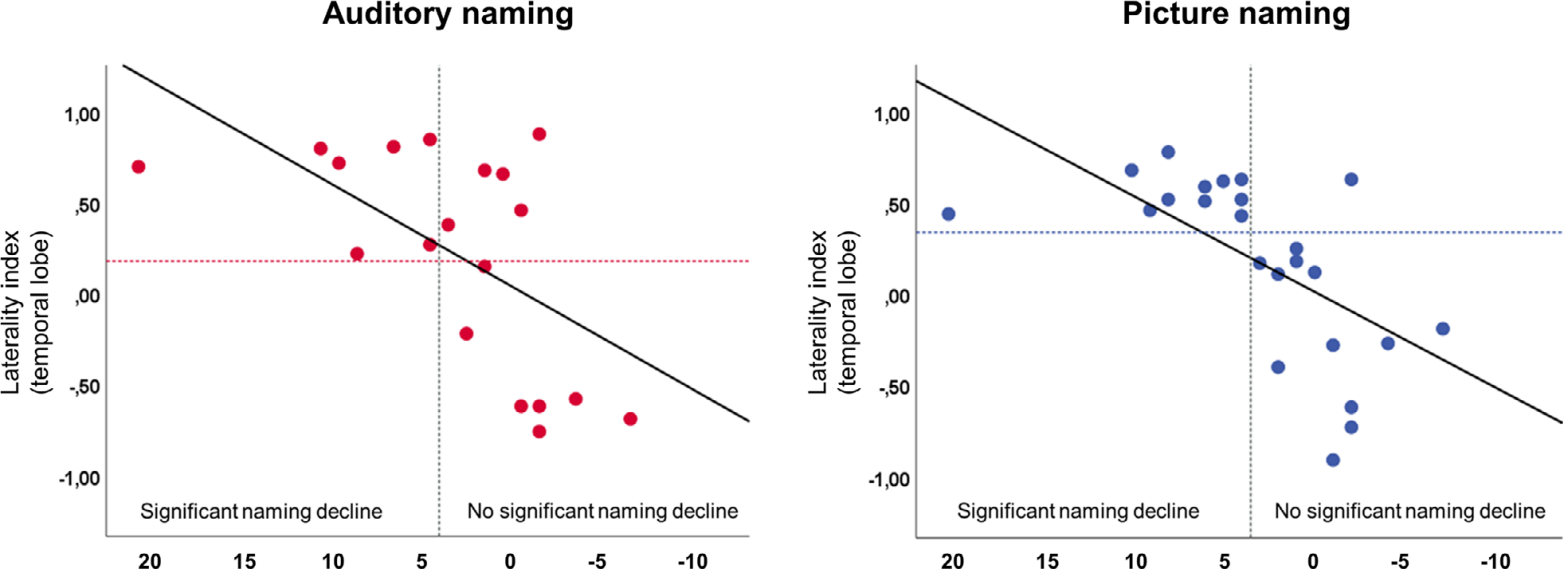
Correlation of laterality indices (LI) of fMRI activations with naming decline in left TLE. A temporal lobe mask was used for the calculation of LIs. Stronger left‐sided lateralization during auditory naming and picture naming was correlated with greater naming decline. For auditory naming (left panel), an LI cutoff value of ≥ 0.18 (red horizontal‐dotted line) identified all patients with a clinically significant naming decline (i.e,. ≥4 points on the McKenna Graded Naming Test). For picture naming (right panel), an LI cutoff value of ≥ 0.34 (blue horizontal‐dotted line) identified all patients with a clinically significant naming decline.

In LTLE patients, stronger left‐sided lateralization correlated with greater naming decline for auditory naming (*ρ* = 0.58; *P* = 0.01; Fig. 3), picture naming (*ρ* = 0.69; *P* < 0.001; Fig. 3), and verbal fluency (*ρ* = 0.45; *P* = 0.03).

In RTLE patients, despite the absence of a relation between the magnitude of activation and naming decline (see subsection 3.4), stronger right‐sided lateralization of picture naming activations correlated with greater naming decline (*ρ* = −0.45; *P* = 0.04). There were no significant correlations of auditory naming or verbal fluency activation lateralization indices with naming decline.

#### Linear regression

In LTLE patients, multiple linear regression showed that, for auditory naming (F(8,10) = 3.04; *P* = 0.049, adjusted *R* ^2^ = 0.44), stronger left‐sided LI was the best predictor of naming outcome (Beta = 0.58, *P* = 0.01, *T* = 2.92), while none of the other parameters investigated (age, age at onset of seizures, gender, preoperative hippocampal volume, preoperative naming score, surgery type) predicted naming decline (*P* > 0.05). For picture naming (F(8,15) = 3.01; *P* = 0.031; adjusted *R* ^2^ = 0.41), again LI was the best predictor (Beta = 0.77, *P* = 0.003, *T* = 3.48), and there was an additional significant contribution of smaller preoperative right hippocampal volume (Beta = −0.73, *P* = 0.03; *T* = −2.47). For verbal fluency, the linear regression model was not significant (F(8,14) = 1.19, *P* = 0.37; adjusted *R* ^2^ = 0.06).

In RTLE, none of the models was significant (auditory naming: F(8,11) = 1.46, *P* = 0.28, adjusted *R* ^2^ = 0.16; picture naming: F(8,11) = 1.74, *P* = 0.19, adjusted *R* ^2^ = 0.24; verbal fluency: F(8,11) = 1.94, *P* = 0.15, adjusted *R* ^2^ = 0.28).

#### Prediction of significant naming decline in individual patients

In LTLE patients, ROC curve analyses for auditory naming yielded an area under the curve (AUC) of 0.82 with a 95% CI between 62% and 100%. A cutoff LI of higher than 0.18 identified all patients with a clinically significant naming decline (Fig. 3), with a positive predictive value (PPV) of 58.3% with 100% sensitivity and 58% specificity. For picture naming, the AUC was 0.94 with a 95% CI between 83 and 100% and an optimal cutoff LI of higher than 0.34 provided a PPV of 91.6% with 100% sensitivity and 92% specificity (Fig. 3, Table 3).

**Table 3:** Picture naming temporal lobe lateralization index (LI) of ≥0.34 predicts clinically significant naming decline (≥4 points on the McKenna Graded Naming Test) after left anterior temporal lobe resection in left temporal lobe epilepsy patients (Chi^2^ = 20.31, *P* < 0.001).

	Significant naming decline (number of LTLE patients)	No significant naming decline (number of LTLE patients)	Total
Picture naming LI ≥ 0.34	11	1	12
Picture naming LI < 0.34	0	12	12
Total	11	13	24

As only three RTLE patients had a clinically significant naming decline, we did not formally perform ROC curve analyses in RTLE. Descriptively, in these three patients, picture naming activations were right‐lateralized in two and bilateral in one patient, auditory naming activations were left‐lateralized in one and bilateral in two, and verbal fluency activations were left‐lateralized in all three patients.

## Discussion

In LTLE patients, stronger auditory and picture naming fMRI activations in the left temporal lobe were related to greater naming decline 4 months following temporal lobe resection, while there was no such effect for frontal lobe activations derived from verbal fluency fMRI. Individual LI analyses revealed that stronger left‐sided lateralization of auditory and picture naming activation in the temporal lobes was the best predictor of greater naming decline, irrespective of age, age at onset of seizures, gender, preoperative naming scores, or surgery type (standard ATLR vs. temporal lobe lesionectomy). Interestingly, for picture naming, a smaller contralateral hippocampal volume was also associated with greater naming decline. Several studies have investigated postoperative plasticity and reorganization of language areas in TLE^5^, ^23^, ^24^ and demonstrated that, particularly in LTLE, language fMRI activation areas can shift to contralateral homologous regions following dominant temporal lobe resection.^1^ One may therefore speculate that a small contralateral hippocampus is a marker of an impaired “reserve” for such reorganization processes, and thus relates to a higher risk of postoperative naming deficits.^25^ We found no association between the age at onset of seizures and postoperative naming performance outcome, which is in accordance with previous investigations.^1^, ^26^ Preoperative naming scores have previously been shown to only weakly correlate with postoperative naming decline in LTLE, and did not withstand multivariate testing.^26^

In RTLE patients, stronger right‐sided lateralization of picture naming activations correlated with greater naming decline, which, however, did not withstand multivariate regression analyses, presumably due to the smaller number of patients with a postoperative naming decline (three of 21 had a clinically significant naming decline). However, it is important to note that none of those three patients with a clinically relevant naming decline was left‐lateralized for picture naming.

### Role of posterior inferior temporal lobe in naming function in TLE

As previously demonstrated,^7^, ^12^ the auditory and picture naming tasks used in this study elicit robust activation in the left posterior inferior temporal lobe, a cortical site that is typically not resected during standard ATLR. Activation in this region is strongly related to better clinical naming function,^7^ which is also in line with cortical stimulation^27^ and lesion studies.^28^ Recently, a combined fMRI, electrocorticography and direct cortical stimulation study demonstrated a typical time course of cortical activation patterns during visual and auditory object naming: primary sensory processing, semantic processing, and articulatory planning, and identified the left fusiform gyrus as a “semantic hub” for lexical semantic processing.^29^

Left posterior temporal lobe naming regions are functionally coupled to the anterior temporal lobe, including the temporal pole, and the intensity of functional connectivity measures between these regions was previously shown to be associated with better clinical naming in TLE.^7^ This substantiates our finding that fMRI‐derived functional activation with naming in the posterior temporal lobe is predictive of naming decline, even when the cortical region showing naming activation is spared. The inference is that naming decline may be the result of partial disconnection of the posterior inferior temporal lobe during ATLR.

### Individual prediction of clinically relevant naming decline

Only a few studies have investigated the potential of fMRI to predict naming decline in individual patients.^1^, ^26^, ^30^, ^31^ In LTLE, an auditory naming temporal lobe LI of >0.18 predicted a clinically relevant naming decline with a PPV of 54% with 100% sensitivity and 58% specificity. Picture naming LI was more specific; an LI of >0.34 gave a PPV of 92% with 100% sensitivity and 92% specificity. A previous study in 44 TLE patients (LTLE and RTLE)^1^ showed that lateralization of frontal lobe activations during verbal fluency fMRI predicted significant naming decline in LTLE, however, with a low specificity of 33.3% (40% when considering both verbal fluency LI and preoperative naming scores). Two studies included the predictive value of temporal lobe fMRI activations, and found that using an auditory semantic decision task, a significant naming decline could be predicted after temporal lobe resection with a PPV of 46%^31^ respectively 57%.^26^ The current study demonstrates that, using picture naming, the specificity of the prediction of naming decline is substantially improved (92% specificity and PPV). The high specificity of both auditory and picture naming may be explained by the design of the fMRI tasks, particularly the use of an active baseline condition, which allows to probe higher order naming function in a more specific manner. The further improved specificity for picture naming may be attributed to the strong bilateral representation of visual‐spatial function^32^ and the differential information processing of auditory and visual information, where the left hemisphere shows a tendency to be the representation of “local details,” whereas the right hemisphere is involved in more “global” representations.^33^ This is reflected in the relatively high proportion of bilateral or right‐sided lateralization of visual naming activations in our cohort, even in healthy controls. Future studies should explore whether the exploration or development of other auditory paradigms may lead to improved specifiticy.

Only three of 21 RTLE patients developed a clinically significant postoperative naming decline, so no formal prediction analyses were carried out. Of note, however, none of these three patients were left‐lateralized for picture naming, and stronger right‐sided lateralization of picture naming was correlated with greater naming decline. These findings point to a potential predictive use of naming fMRI tasks in RTLE as well, and future studies with larger samples are warranted to confirm these findings.

Predicting the risk of postoperative naming decline is of utmost importance for epilepsy patients, since word finding difficulties represent the most relevant cognitive impairment by patients after temporal lobe surgery^34^ and cause intense feelings of frustration, embarrassment, and inadequacy. Word finding difficulties put individuals at a considerable disadvantage in formal settings, such as job interviews, but also cause difficulties in less formal social gatherings, with consequent loss of self‐confidence and higher tendency to avoid mixing with others. The ensuing social isolation can in turn trigger low self‐esteem and low mood. A decline of ≥ 4 points on the GNT is considered very clinically relevant, and sensitive to detect declines over time.^10^

### Strengths and limitations

We used overt auditory and picture naming tasks, which allowed active control for task performance, and directly compared their prediction of naming decline to a standard covert verbal fluency task, which is widely applied clinically in routine presurgical language assessment. Furthermore, the two overt naming tasks included active baseline conditions, which allowed to negate activation caused by the type of stimulus presentation (auditory, picture), primary speech processing as well as motor cortex activations, and movement artifacts caused by overt speech production.^12^ This led to display of higher order naming function activations in the posterior basal temporal lobe, which resulted in high specificity and PPV to predict naming decline, particularly for picture naming.

Naming ability was assessed 4 months after temporal lobe resection, and reorganization may continue over a longer timeframe. We are currently undertaking longitudinal follow‐up assessments over 12 months after temporal lobe resection, including repeat fMRI measurements to address issues of postoperative plasticity. Postoperative naming outcome was assessed using a visual confrontation naming test, which is widely used in clinical practice.^35^, ^36^ However, it has been suggested that performance on auditory naming tasks seems to be particularly related to word finding difficulties in conversational speech with its multiple neuropsychological demands,^37^ which should be addressed in future studies.

A number of subjects were excluded due to technical problems or floor effects, and we cannot ascertain whether findings in those subjects could affect the primary results. Approximately half of our patients had hippocampal sclerosis, but other patient groups, such as FCD or DNT were also included. Subgroup analyses were not performed due to insufficient sample sizes. It is important to note that all patients had identifiable lesions on MRI, which can positively affect seizure and cognitive outcomes.^38^ In future studies with larger sample sizes including MR‐negative patients, further effects of etiology as well as potential discrepancies between adult and pediatric populations should be explored.

Although drug load was comparable in LTLE and RTLE patients, we did not account for a potential effect of medication on fMRI activations. Topiramate and zonisamide may particularly affect language fMRI activation patterns,^39^, ^40^ which should be addressed in future investigations. It is important to note, however, that the number of patients taking topiramate or zonisamide as well as the respective daily doses were comparable between LTLE and RTLE patients.

### Clinical implications

While temporal lobe resection represents an effective treatment option for refractory TLE, leading to seizure remission in up to 80% of patients,^41^ the risk of postoperative naming and word finding deficits is a major concern. We show that auditory and, particularly, picture naming fMRI can specifically predict naming decline in LTLE following temporal lobe resection. Furthermore, we also provide the first evidence for a potential use in RTLE patients. This has implications for the implementation of auditory and picture naming fMRI into routine presurgical protocols, to further aid surgical planning and help to mitigate postoperative naming deficits.

## Author Contributions

KT, PJT, and JSD formulated the study design. KT, LAG, GGG, AH, and LC acquired imaging data. LC and SV contributed to imaging data analysis. KT carried out the image processing and statistical analyses of neuropsychological and imaging data, interpreted the data, wrote the manuscript, and prepared all the supporting material. All authors contributed to data interpretation and manuscript preparation. KT, LC, and PJT acquired neuropsychological data. PJT, MJK, and JSD supervised data analysis, interpretation, and manuscript preparation. All authors approved the final version of the manuscript before submission.

## Conflict of Interest

Nothing to report.

## Supporting information


**Table S1.** MNI Coordinates and Z‐scores of whole brain cluster‐level activations and deactivations across all subjects (LTLE, RTLE, controls) during auditory naming, picture naming, and verbal fluency shown corrected for multiple comparisons (FWE; *P* < 0.05).
**Table S2.** Coordinates and Z‐scores of correlations of fMRI activation during auditory and picture naming with naming decline in left TLE patients, shown at *P* < 0.001 uncorrected masked for the group activation maps.
