# Supplementary material for: Naming fMRI predicts the effect of temporal lobe resection on language decline
Source: Ann Clin Transl Neurol. 2019 Oct 2;6(11):2186–96. doi: 10.1002/acn3.50911 (PMC6856622; doi:10.1002/acn3.50911)
Supplement: Supplementary file 1 — Table S1. MNI Coordinates and Z‐scores of whole brain cluster‐level activations and deactivations across all subjects (LTLE, RTLE, controls) during auditory naming, picture naming, and verbal fluency shown corrected for multiple comparisons (FWE; P < 0.05). Table S2. Coordinates and Z‐scores of correlations of fMRI activation during auditory and picture naming with naming decline in left TLE patients, shown at P < 0.001 uncorrected masked for the group activation maps. [file ACN3-6-2186-s001.docx]

**Appendix**

Supplementary Table 1. MNI Coordinates and Z-scores of whole-brain cluster-level activations and deactivations across all subjects (LTLE, RTLE, controls) during auditory naming, picture naming and verbal fluency shown corrected for multiple comparisons (FWE; p < 0.05).

| **Whole-brain activations** | | | | | | | | | | | | |
| --- | --- | --- | --- | --- | --- | --- | --- | --- | --- | --- | --- | --- |
|  | **Auditory naming** | | | | **Picture naming** | | | | **Verbal fluency** | | | |
|  | **Left  Hemisphere** | | **Right  Hemisphere** | | **Left  Hemisphere** | | **Right  Hemisphere** | | **Left Hemisphere** | | **Right  Hemisphere** | |
|  | Z | Coordinates | Z | Coordinates | Z | Coordinates | Z | Coordinates | Z | Coordinates | Z | Coordinates |
| Inf front G | 6.04 | -40 30 16 |  |  |  |  |  |  | >8 | -46 24 22 |  |  |
| Sup front G | 5.82 | -10 -64 2 |  |  |  |  |  |  |  |  |  | |
| Suppl motor | 5.26 | -6 22 48 |  |  | 4.78 | -4 16 54 |  |  | >8 | -2 10 64 |  | |
| Precentral G |  |  |  |  |  |  |  |  | 7.46 | -54 0 46 |  | |
| Inf temp G | 5.53 | -50 -52 -16 |  |  |  |  |  |  |  |  |  | |
| Mid temp G | 5.04 | -50 -42 -4 |  |  |  |  |  |  |  |  |  | |
| Fusiform G | 5.19 | -44 -48 -20 |  |  | 7.23 | -55 -56 -14 |  |  |  |  |  | |
| Lingual G | 6.14 | -10 -42 -6 |  |  |  |  |  |  |  |  | 4.72 | 18 -88 -8 |
| Inf par lob |  |  |  |  |  |  |  |  | 4.76 | -44 -44 40 |  |  |
| Inf occipit G |  |  |  |  |  |  | >8 | 42 -78 -8 | 6.05 | -38 -82 -10 |  | |
| Mid occip G |  |  |  |  | 7.81 | -40 -74 -8 |  |  |  |  |  |  |
| Sup occip G | 5.93 | -10 -86 8 |  |  | 4.57 | -12 -80 6 | 5.60 | 12 -82 8 |  |  |  | |
| Cerebellum |  |  |  |  |  |  | 7.35 | 28 -42 -26 | 6.65 | -40 -64 -30 | >8 | 32 -64 -30 |
|  |  |  |  |  |  |  |  |  |  |  |  |  |
| **Whole-brain deactivations** | | | | | | | | | | | | |
|  | **Auditory naming** | | | | **Picture naming** | | | | **Verbal fluency** | | | |
|  | **Left  Hemisphere** | | **Right  Hemisphere** | | **Left  Hemisphere** | | **Right  Hemisphere** | | **Left Hemisphere** | | **Right  Hemisphere** | |
|  | Z | Coordinates | Z | Coordinates | Z | Coordinates | Z | Coordinates | Z | Coordinates | Z | Coordinates |
| Precuneus |  |  | 7.60 | 4 -74 38 |  |  | 7.17 | 4 -64 36 |  |  | >8 | 6 -60 34 |
| Angular G | 6.28 | -40 -70 50 |  |  | 5.61 | -42 -70 48 | 7.10 | 42 -78 40 | 7.45 | -46 -64 24 |  |  |
| Cingulate |  |  |  |  | 5.62 | -8 -44 34 | 6.51 | 10 -50 30 |  |  | >8 | 6 -52 30 |
| Supramar G |  |  | 6.51 | 42 -48 38 |  |  |  |  |  |  |  |  |
| Inf par lob | 6.57 | -60 -50 42 |  |  |  |  |  |  |  |  | 5.60 | 56 -26 24 |
| Mid front G | 4.90 | -30 50 2 | 5.48 | 40 36 38 |  |  | 5.41 | 46 20 50 |  |  | 5.08 | 26 32 40 |
| Sup front G | 4.77 | -20 54 0 | 6.03 | 28 18 60 |  |  |  |  |  |  |  |  |
| Orbitofront G |  |  |  |  |  |  |  |  | 7.16 | -6 38 -10 | 7.59 | 2 60 -2 |
| Precentral G |  |  |  |  |  |  |  |  |  |  | 6.75 | 10 50 -12 |
| Sup temp G | 5.03 | -50 -6 -2 |  |  |  |  |  |  |  |  |  |  |
| Mid temp G |  |  |  |  | 6.61 | -56 -68 26 | 5.21 | 44 6 -42 | 5.33 | -56 -12 -20 | 7.33 | 50 -62 16 |
| Inf temp G |  |  |  |  | 4.55 | -54 0 -34 | 4.57 | 56 -30 -20 | 5.60 | -52 -2 -34 |  |  |
| Mid occip G |  |  |  |  | 6.36 | -40 -80 40 |  |  |  |  | 7.33 | 46 -74 28 |

Note: Front = frontal; G = gyrus inf = inferior; L = left; lob = lobule; med = medial; mid = middle; MNI= Montreal Neurological Institute; par = parietal; occip = occipital; orbitofront = orbitofrontal; R = right; sup = superior; supramar = supramarginal; suppl motor = supplementary motor region; temp = temporal; TLE = temporal lobe epilepsy.

Supplementary Table 2. Coordinates and Z-scores of correlations of fMRI activation during auditory and picture naming with naming decline in left TLE patients, shown at p < 0.001 uncorrected masked for the group activation maps.

| **Naming decline** | | | | | | | | |
| --- | --- | --- | --- | --- | --- | --- | --- | --- |
|  | **Auditory naming** | | | | **Picture naming** | | | |
|  | **Left** | | **Right** | | **Left** | | **Right** | |
|  | Z | Coordinates | Z | Coordinates | Z | Coordinates | Z | Coordinates |
| Inf front G | 3.61 | -52 32 8 |  |  |  |  |  |  |
| Inf temp G | 3.57 | -46 -60 -10 |  |  |  |  |  |  |
| Fusiform G |  |  |  |  | 3.32 | -30 -62 -16 |  |  |
| Cerebellum |  |  |  |  |  |  | 4.19 | 8 -56 -36 |
| Mid occip G |  |  |  |  | 3.52 | -26 -64 -36 |  |  |

Note: Front = frontal; G = gyrus inf = inferior; mid = middle; occip = occipital; temp = temporal; TLE = left temporal lobe epilepsy.
